# Compréhension du mode de transmission des agents du paludisme: une étonnante suite d'hypothèses, d'observations, de recherches et de controverses

**DOI:** 10.48327/mtsi.v3i1.2023.312

**Published:** 2023-02-13

**Authors:** François Rodhain

**Affiliations:** Professeur honoraire à l'Institut Pasteur; SFMTSI Société francophone de médecine tropicale et santé internationale (ancienne SPE), Hôpital Pitié-Salpêtrière, Pavillon Laveran, 47-83 Boulevard de l'Hôpital, 75651 Paris cedex 13, France; * Actes du Colloque – Centenaire de la mort d'Alphonse Laveran. 24 novembre 2022, Paris / Proceedings of the Conference – Centenary of the death of Alphonse Laveran. 24 November 2022, Paris

**Keywords:** Alphonse Laveran, Ronald Ross, Paludisme, Histoire du paludisme, *Plasmodium*, *Anopheles*, Moustique, Vecteur, Alphonse Laveran, Ronald Ross, Malaria, History of malaria, *Plasmodium*, *Anopheles*, Mosquito, Vector

## Abstract

En 1880, Alphonse Laveran observait l'agent causal du paludisme. Dès 1884, il considéra que des moustiques pourraient être responsables de la transmission des hématozoaires, hypothèse qui résultait de l'observation et de la réflexion d'un hygiéniste averti. Mais, comme le dit Laveran lui-même, « l'opinion que je défendais était considérée par la plupart des observateurs, comme très peu vraisemblable ».

Près de 15 ans après la découverte de l'hématozoaire, l’élucidation du mécanisme de transmission s'avérait toujours difficile à établir. Certes, un lien avec l'existence de marécages avait été établi de longue date, mais le véritable mode de transmission demeura un mystère jusqu’à la fin du XIX^e^ siècle. L'implication, par Manson en 1877, des moustiques dans le cycle de la filaire de Bancroft, puis d'autres observations du même ordre, finirent par attirer l'attention des malariologistes. Laveran lui-même fut rapidement convaincu du rôle des moustiques dans la réalisation du cycle naturel et la propagation des *Plasmodium.* Toutefois, cette théorie avait autant de détracteurs que de soutiens.

En 1897, Ross montra la présence d'oocystes sur l'estomac de moustiques préalablement gorgés sur un malade paludéen, puis en 1898, de sporozoïtes de plasmodies d'oiseaux chez les moustiques. Il était persuadé que, par leur piqûre, ces insectes étaient responsables de la transmission des agents du paludisme humain, sans pouvoir en apporter la preuve. Les résultats obtenus par Ross furent aussitôt confirmés en Italie par Grassi et ses collaborateurs qui, en novembre 1898, décrivirent les stades des *Plasmodium* de l'Homme et, par différentes expériences menées en collaboration avec les chercheurs britanniques, montrèrent le rôle des anophèles, ce qui fut loin d’être accepté par tous. Le scepticisme persista longtemps.

Excellent protozoologiste, Laveran n’était pas entomologiste. Il fut cependant parmi les premiers défenseurs de la théorie anophélienne. Il travailla beaucoup à l’établissement des rapports entre anophèles et paludisme et s'intéressa de près aux conditions environnementales de la transmission. Dans son esprit, la fièvre palustre devait être classée désormais parmi les maladies évitables. Une ère d'espoir s'ouvrit alors: la prophylaxie du paludisme, basée sur la défense contre les moustiques, pouvait commencer.

## Abstract (en)

In 1880, Laveran observed the causative agent of malaria. As early as 1884, he considered that mosquitoes could be responsible for the transmission of haematozoa, a hypothesis which resulted from the observation and reflection of an informed hygienist. But, as Laveran himself said, “the opinion that I defended was considered by most observers to be highly unlikely”.

Nearly 15 years after the discovery of the haematozoan, the elucidation of the mechanism of transmission still proved difficult to establish. A link with the existence of swamps had been established a long time before, but the true mode of transmission remained a mystery until the end of the 19th century. The implication, by Manson in 1877, of mosquitoes in the cycle of the Bancroftian filaria, then other observations of the same order, ended up attracting the attention of malariologists. Laveran himself was quickly convinced of the role of mosquitoes in carrying out the natural cycle and propagating *Plasmodium*, but this theory had as many detractors as supporters.

In 1897, Ross showed the presence of oocysts on the stomach of mosquitoes previously gorged on a malaria patient, then in 1898, of sporozoites of bird plasmodia in mosquitoes. He was convinced that, through their bite, these insects were responsible for the transmission of human malaria agents, without being able to prove it. The results obtained by Ross were immediately confirmed in Italy by Grassi and his collaborators who, in November 1898, described the stages of *Plasmodium* in man and, through various experiments carried out in collaboration with British researchers, showed the role of *Anopheles*, a result far from being accepted by all. Skepticism persisted for a long time.

An excellent protozoologist, Laveran was not an entomologist. He was however among the first defenders of the anopheline theory. He worked extensively on establishing the relationships between *Anopheles* mosquitoes and malaria and took a close interest in the environmental conditions of the transmission. In his mind, malaria fever should henceforth be classified as a preventable disease. An era of hope thus dawned: malaria prophylaxis, based on fight against mosquitoes, could begin.

## Introduction

**6 novembre 1880.** Dans l'histoire des sciences médicales, ce jour marque une avancée de toute première importance: l'observation par Alphonse Laveran, en Algérie, d'un protozoaire parasite qu'il nomme *Oscillaria malariae* et qu'il considère d'emblée comme l'agent du paludisme. Une plaque a longtemps été apposée dans l'hôpital de Constantine (elle s'y trouve peut-être encore) pour rappeler cette découverte majeure dans l'histoire de nos connaissances en parasitologie, en médecine tropicale, et en médecine tout court.

**20 août 1897 (« *mosquito day* »).** Ronald Ross, à Secunderabad en Inde, observe, sur la paroi de l'estomac de moustiques préalablement gorgés sur un malade paludéen, ce que nous appelons aujourd'hui des oocystes. Il pressent qu'il s'agit d'un stade particulier du développement du même parasite sans toutefois pouvoir le prouver. Il y a quelques années (c'est peut-être encore le cas aujourd'hui), lorsqu'on pénétrait dans la bibliothèque de la London School of Tropical Medicine à Londres, on trouvait sur sa droite, sur un trépied, le cahier de laboratoire de Ross ouvert à la page de cette date mémorable. Ce n'est que près d'un an plus tard, en juillet 1898, que Ross, travaillant sur des plasmodies d'oiseaux, peut observer des sporozoïtes et comprend le mécanisme de la transmission de ces parasites. Ces résultats sont confirmés en novembre 1898 par les expériences décisives des savants italiens Grassi, Bastianelli et Bignami qui décrivent le cycle de développement et la transmission des *Plasmodium* humains par les anophèles.

Dix-huit années se sont donc écoulées entre ces deux découvertes, 18 années de recherches et d'hésitations durant lesquelles d'innombrables observations, de très nombreuses expériences ont été réalisées, constamment accompagnées de discussions passionnées, de scepticisme persistant et de controverses.

Quelles étaient les idées de Laveran dans ce contexte particulier?

## Étiologie Des Fièvres Palustres Avant Laveran: Les Hypothèses

Bien des savants avaient cherché, depuis des siècles, à identifier un « microbe » susceptible d’être responsable des fièvres palustres. Le lien, établi de longue date en Europe avec les marécages, lien sur lequel nous reviendrons plus bas, amenait régulièrement nombre de scientifiques à rechercher dans les eaux stagnantes l'agent causal du paludisme; on a incriminé tour à tour des émanations néfastes provenant de l'eau, de la vase ou de l'air des marais, puis des miasmes, des animalcules (notamment Jérôme Fracastor au xvi^e^ siècle ou Giovanni Maria Lancisi au xviii^e^ siècle), enfin des micro-organismes: des champignons comme *Limnophysalis hyalina* pour le Suédois Fredrik Eklund en 1878, ou encore des algues d'eau douce comme des *Palmella* pour l'Américain J. H. Salisbury en 1866, ou *Alga miasmatica* pour l'Italien Pietro Balestra en 1869 et 1877; des bactéries ont aussi été soupçonnées, comme le *Bacillus* (ou *Bacteridium) brunneum* de Matteo Lanzi et Guglielmo Terrigi en 1876, ou encore le *Bacillus malariae* trouvé par Edwin Klebs et Corrado Tommasi-Crudeli dans la vase en 1878-79, dernière hypothèse qui fut un temps soutenue par des sommités comme Ettore Marchiafava, Angelo Celli, Edoardo Perroncito et bien d'autres.

Une étiologie bactérienne spécifique, avec un réservoir essentiellement hydrique, semblait évidente aux yeux de beaucoup, confortée par la recrudescence de la maladie lors de pluies abondantes, inondations ou débordement des rivières [18]. Mais, comme le dit très justement Manson en 1882: « Nous sommes dans l’ère des bactéries; elles sont recherchées partout et dans toutes les maladies; elles sont donc trouvées partout et dans toutes les maladies. » Et Valentiny ajoute: « À cultiver ou à inoculer à partir de l'air, de l'eau ou des vases des marais, le prodige eût été d'obtenir des résultats négatifs [32]. » Marie Phisalix souligne que « si Laveran avait seulement suivi la même voie, il n'aurait pas été plus heureux que ses prédécesseurs, pour l'excellente raison que le microbe n'existe à l'air libre dans aucun de ces milieux [22] ».

Se posait également la question de l'identité du pigment palustre. En effet, bien avant Laveran, certains pathologistes comme Lancisi en 1718, Maillot en 1836, Heinrich Meckel en 1847 et bien d'autres, remarquant, lors d'autopsies, la teinte foncée des viscères, avaient vu l'accumulation du pigment palustre dans les organes (foie, cerveau, cellules du sang) ou même sans doute dans les parasites eux-mêmes sans se rendre compte de leur nature; ce fut le cas pour Frerichs en 1858 ou encore pour Kelsch en 1875. Rudolf Virchow, lui aussi, avait remarqué ce pigment en 1848 mais il avait pris les corps pigmentés pour des leucocytes (il y a certes des leucocytes mélanifères) et, à l’époque, personne ne pouvait se permettre de discuter Virchow !

## 1880: La Découverte De Laveran. Contexte Et Controverses

Mais venons-en aux travaux de Laveran. Dans la conduite de ses travaux, il ne partit pas, comme la plupart des chercheurs d'alors, de prélèvements de sols, d'eaux ou de vase ou d'autres éléments de l'environnement mais de l'anatomie pathologique et du sang des malades. Après de premiers travaux dans les hôpitaux de Bône puis de Biskra, c'est à l'hôpital de Constantine que Laveran observa, le 6 novembre 1880, dans le sang d'un malade fiévreux, un curieux micro-organisme qu'il nomma *Oscillarla malariae* et qu'il considéra d'emblée comme l'agent causal du paludisme [30]. Cette conviction, Laveran l'exprima d'abord en deux notes successives communiquées à l'Académie de médecine le 23 novembre et le 28 décembre 1880 [17].

Et pourtant, la question n’était pas aussi simple qu'il paraît *a priori.*

Laveran profita de la chance dans la mesure où, observant des gamétocytes, il fut le témoin d'un phénomène inattendu: l'exflagellation, c'est-à-dire la formation d’éléments flagellés très mobiles, un phénomène qui se produit habituellement chez le moustique vecteur^1^1Il semblerait que ce soit Paul-Louis Simond qui a émis l'idée que les flagelles de l'hématozoaire étaient des éléments mâles destinés à féconder des éléments femelles.. Il en déduisit, à l’étonnement général, qu'il s'agissait d'un protozoaire parasite et non d'une bactérie comme beaucoup s'y attendaient. Certes, ce n’était pas la première fois qu'un protozoaire se trouvait impliqué dans l’étiologie d'une maladie de l'homme; cinq ans auparavant, Friedrich Lösch avait décrit, chez des patients russes, *Entamoeba histolytica* (qu'il avait alors dénommé *Amoeba coli)*, et montré son rôle comme agent de la dysenterie amibienne. Mais, rapidement, Metchnikoff montra que l'hématozoaire de Laveran n'appartenait pas aux Rhizopodes, comme c’était le cas pour les amibes de Lösch, mais aux Sporozoaires.

Toutefois, la démonstration de l'implication du parasite en question dans l’étiologie des fièvres palustres n'allait pas de soi. Si, dans ce contexte éminemment concurrentiel, de grands noms, comme Sternberg, Osler, Metchnikoff, Councilman, James, etc., ont rapidement soutenu les idées de Laveran, cette découverte ne fut pas facilement acceptée par tous et suscita beaucoup de scepticisme. À l'Académie de médecine, Léon Colin qui, en 1870, avait rédigé un *Traité des fièvres intermittentes*, doutait fortement du rôle pathogène des corpuscules décrits par Laveran. Pasteur lui-même n'y croyait guère, de même que Laboulbène. Émile Duclaux également, qui admettait encore l'existence du *B. malariae* de Klebs et écrivait, dans son ouvrage *Ferments et maladies* de 1882: « On peut se hasarder à dire que si M. Laveran a bien observé, il a moins bien compris la signification des faits qui lui passaient sous les yeux et que chez ces malades, c'est encore un bacillus qui est l'espèce active [10]. » De son côté, Raphaël Blanchard, dans son *Traité de zoologie médicale* de 1889, ne mentionnait pas l'hématozoaire de Laveran parmi les Protozoaires parasites de l'homme. En réalité, les contradicteurs reprochaient surtout à Laveran de ne pas s’être plié au mode d'expérimentation pasteurien qui supposait une mise en culture du germe et une reproduction de la maladie par inoculation à l'animal [19]. Et ce malgré d'innombrables essais, de la part de Laveran et d'autres, tous demeurés infructueux, et pour cause ! C'est bien ce que regrette Corre dans l'analyse qu'il donne du *Traité des fièvres palustres avec la description des microbes du paludisme* de Laveran, où il écrit en 1884 *(Archives de médecine navale*, p. 418): « Cette doctrine [le protozoaire parasite comme cause du paludisme] est exposée et défendue avec une grande modération de forme, qui n'exclut pas la vigueur dans l'argumentation, en même temps qu'avec une conviction réelle et une incontestable habileté. Cependant, notre distingué confrère n'a pas réussi à dissiper, selon nous, les objections que nous lui avons faites à diverses reprises […]. Monsieur Laveran ne le peut démontrer ni par la méthode des cultures, ni par celle des inoculations. »

Quant aux savants italiens, en premier lieu Marchiafava et Celli, très impliqués dans cette recherche, ils mettaient fortement en doute le rôle de l'hématozoaire. Mais tous deux furent finalement convaincus par Laveran qui entreprit, à cet effet, le voyage de Rome en 1882. Un peu plus tard, Pasteur, Roux et Chamberland reconnurent la justesse des conclusions de Laveran qui leur montrait « les corps pigmentés, les croissants et les flagelles ».

La situation s'est ensuite compliquée lorsqu'il parut probable que le parasite de Laveran n’était pas la seule espèce en cause dans les paludismes humains. Cela constituait une difficulté supplémentaire: plusieurs espèces différentes pouvaient intervenir, ce qui suppose l'existence éventuelle de plusieurs cycles d'inégale durée. Or, qu'une même maladie revendiquât plusieurs agents causaux était un fait encore sans exemple. En 1885, Bretonneau estimait qu’« un germe spécial propre à chaque contamination donne naissance à chaque maladie contagieuse ». Laveran a toujours soutenu la doctrine de l'unicité du parasite du paludisme. « Les différentes espèces d'hématozoaires admises par un grand nombre d'auteurs ne sont pour lui que des variétés susceptibles de se transformer l'une dans l'autre [22]. » Laveran écrit: « Les différences morphologiques qui existent entre les parasites des fièvres tropicales et ceux des fièvres tierces ou quartes me paraissent devoir être rapportées à des variétés d'un même hématozoaire et non à des espèces distinctes [22]. »

C'est quelques années plus tard, à partir de 1885-86, que Camillo Golgi montre, à Pavie, sur des paludéens de la plaine du Pô, le synchronisme du cycle parasitaire, que ce synchronisme est différent pour les fièvres tierces et les fièvres quartes, et que les pics fébriles sont dus à la croissance et la multiplication des formes parasitaires dans les globules rouges envahis par l'hématozoaire; ce qui l'amène à considérer l'existence de deux espèces de Plasmodies: *Plasmodium vivax* et *P. malariae*, ce dont doutaient de nombreux médecins et Laveran lui-même qui écrit: « La pluralité des parasites du paludisme [n'est] pas démontrée et l'existence d'un seul hématozoaire polymorphe [est] au contraire probable [22]. » En 1889-90, Grassi et Feletti donnent une description de *P. vivax*; la même année, Marchiafava, Bignami, Celli et leurs collaborateurs observent *P. falciparum* dans les marais Pontins (l'espèce sera décrite par Welch en 1897) et montrent la sévérité de l'infection par ce parasite. Grassi et Feletti établissent finalement l'existence de trois espèces^2^2On peut lire dans *La Presse médicale* de 1907 (p. 332-333) les lignes suivantes: « Des controverses naquirent sur la question de savoir s'il fallait ajouter aux trois variétés parasitaires acceptées par les Italiens de nouvelles variétés ou, au contraire, comme n'avait cessé de le prétendre Laveran, s'il convenait de les ramener toutes à une seule forme, dont les autres ne constitueraient que des variations morphologiques adaptées au climat, aux qualités individuelles de l'organisme infecté, etc. C'est ainsi que R. Koch a cru devoir en décrire une quatrième; Schaudinn, allant plus loin encore, admettait une douzaine de variétés parasitaires en tout. ». L'espèce *Plasmodium ovale* ne sera décrite qu'en 1922 par Stephens.

Quoi qu'il en soit, la découverte de Laveran devait fait entrer le paludisme dans la théorie infectieuse des maladies de Pasteur. Elle mettait fin au rôle de facteurs « telluriques » ou « miasmatiques » qui circulaient jusqu'alors. « Laveran a su faire triompher, contre vents et marées, une révélation balayant toutes idées reçues [32] ». Comme Émile Roux lui dit bien plus tard, en 1915: « À bien réfléchir, je trouve que vous n'aviez pas lieu de vous plaindre. Apportant une chose aussi neuve, vous méritiez d’être encore plus malmené. »

Le parasite responsable du paludisme humain étant identifié, restait à en comprendre le mécanisme de transmission. Nouvelles recherches, et surtout nouvelles controverses en perspective.

## Transmission Des Agents Du Paludisme: Les Hypothèses AntéRieures Aux Travaux De Manson

Sans remonter à la citation classique des écrits des savants indiens, en particulier Sushruta de Bénarès dont le texte sanscrit est difficile à interpréter et dont les dates sont des plus incertaines, on sait bien que, dans le bassin méditerranéen, les Grecs et les Romains avaient établi une relation entre eaux stagnantes et « fièvres ».

Pour ce qui est des Grecs, on retiendra notamment que le grand Hippocrate (env. 460-377 av. J.-C.), le premier malariologiste selon Russell [28], dans son fameux traité *Des airs, des eaux et des lieux*, pièce maîtresse du Corpus hippocratique, établit clairement un lien entre splénomégalie et marais: « Ceux qui en font usage [qui boivent l'eau des marais] ont toujours la rate volumineuse et dure, le ventre émacié et chaud, les épaules et les clavicules décharnées. […] Pendant l’été, les habitants des régions aux eaux dormantes, aussi bien des marais que des étangs, sont atteints de dysenterie, de diarrhées, de fièvre quarte de longue durée. » *P. vivax* et *P. malariae* existaient en Grèce au v^e^ siècle avant J.-C. Hippocrate, de même que Celse (Aulus Cornelius Celsus, – 25 à + 50) dans son *De Medicina* et Galien (Claudius Galenus, 129-216), avaient bien remarqué la saisonnalité des « fièvres des marais ».

Les Latins avaient donc, eux aussi, très bien établi le lien avec les marais (on retrouve cette notion dans les écrits de Juvénal, de Pline, de Cicéron, de Sénèque, etc.). Varron (Marcus Terentius Varro, 116-27 av. J.-C.) précise bien, dans son *Traité sur l'Agriculture* en 37 av. J.-C., quel doit être l'emplacement d'une ferme, qui ne doit pas être exposée aux vents infectés provenant des marais mais installée sur une hauteur et ensoleillée – « Ainsi les petites créatures que notre œil ne peut voir et qui passent par l'air et dans notre corps par la bouche et nos narines, sont emportées ou meurent desséchées. ») Il en est de même de l'architecte Vitruve qui, au I^er^ siècle avant J.-C., évoque dans son *De Architectura* un rôle possible de la faune des marais, ou encore de Columelle qui indique dans *De Re Rustica*: « Les marais soufflent une odeur nauséabonde funeste lorsqu'il fait chaud et produisent des insectes piqueurs qui attaquent en essaims denses. » Pour la majorité des savants de l’époque, c'est bien l'air putride qui s'exhale des marécages et les miasmes qu'il contient, qui doivent être accusés, mais il n'est pas question des moustiques en tant qu'agents propagateurs.

Les Grecs et les Romains étaient donc bien au courant des effets délétères des marais qu'ils s'efforçaient de maîtriser au moins partiellement par des drainages, le choix des sites de construction des maisons et des règlements précis. Empédocle, un philosophe pré-socratique grec d'Agrigente (Sicile) du v^e^ siècle avant J.-C., aurait protégé la ville de Sélinonte en éliminant les marais et en détournant deux rivières coulant à proximité. À Rome, les drainages ont été très développés; la *Cloaca Maxima* (qui asséchait le marécage situé entre le Quirinal, l'Esquilin et le Tibre et qui fonctionne toujours) daterait du vi^e^ siècle avant J.-C., et des conduites enterrées en terre cuite ont sans doute joué un rôle important dans le contrôle des maladies transmissibles.

Ce lien entre les épidémies de « fièvres » et les eaux stagnantes perdura dans toute l'Europe jusqu'au xix^e^ siècle. Ce rapprochement est rappelé, entre autres, par Avenzoar (1092-1162) dans le califat de Cordoue, en Espagne, mais il existe aussi dans de nombreuses régions françaises: plaine orientale de la Corse (région d'Aléria), Camargue (en particulier Aigues-Mortes et Marignane) où la situation du paludisme sera plus tard si bien évoquée par Alphonse Daudet dans ses *Lettres de mon moulin*, textes qui rappellent parfaitement les descriptions des fièvres palustres données par Hippocrate.

Au xii^e^ siècle, les moines Bénédictins, en particulier les Cisterciens dont l'abbaye est fondée à Cîteaux (Cistercium) en 1098 sur des terres considérées comme malsaines, font preuve d'un véritable génie hydraulique et mettent en valeur des terres en asséchant les marais^3^3De même, dans la vallée de l'Aude, les Cisterciens supprimèrent une vaste région marécageuse d'où le nom de « Clairvaux » donné à leur abbaye, ou encore sur le site de l'abbaye Notre-Dame de Port Royal des Champs, d'abord établie au xiii^e^ siècle en vallée de Chevreuse, « dans un vallon humide et insalubre propice au paludisme ».. À l'inverse, le grand développement des étangs de pisciculture au Moyen Âge et à la Renaissance entraîne une recrudescence du paludisme.

À la Renaissance, l'Italie est la « péninsule des fièvres » avec des foyers très importants dans la plaine du Pô, dans la Maremme en Toscane et, bien sûr, les marais Pontins. À la fin du xix^e^ siècle, le paludisme, toujours endémique en Italie, occasionnait encore quelque 15 000 morts par an et avait un impact socio-économique dramatique.

Aux Pays-Bas, région hautement endémique, la relation entre épidémies de fièvres et marais côtiers est établie de longue date, par exemple par Herman van der Heyden en 1645: « Cette infection est causée d'une maligne et puante vapeur ce qui arrive ordinairement aux-dicts polder's. »

Linné lui-même estime, dans sa thèse de médecine soutenue en 1735, que les fièvres intermittentes seraient dues à des miasmes provenant des régions marécageuses.

Aux xvii^e^ et xviii^e^ siècles, on pensait donc toujours que les fièvres étaient liées à l'air pollué, vicié, des marais généré par la vase chaude (d'où « mal'aria »)^4^4Le terme aurait été utilisé pour la première fois par Sansovino en 1560, mais peut-être déjà par Jacquier en 1743, et dans la langue anglaise dès 1827 semble-t-il, mais ce ne serait quen 1878 que, dans la littérature italienne, ce mot désigne la maladie (dans *La malaria di Roma* de Guido Bacelli)., ou bien à l'eau stagnante des marais (d'où « paludisme »). Selon les auteurs, les miasmes entraient dans le corps par inhalation, par l'eau de boisson contaminée ou par contact.

Il convient toutefois de se pencher ici sur les écrits de Giovanni Maria Lancisi (1654-1720). Dans son ouvrage *De noxiis paludum effluviis, eorumque remedia*, publié à Venise en 1717, il évoque, à propos de la fièvre des marais, le rôle d'un « poison » constitué d'animalcules émanant des marais et transmis par les « insectes des marais^5^5Lancisi était un épidémiologiste reconnu. En 1715, il avait déjà donné des recommandations tout à fait judicieuses au pape Clément XI dont il était le camérier (ou camerlingue: au service personnel du pape) pour prévenir l'extension de la peste bovine aux États pontificaux. ». Mentionnons également Giovanni Rasori (1766-1837), de Pavie, à la toute fin du xviii^e^ siècle qui parle d'un parasite dont le cycle reproductif serait responsable des attaques successives et pense aux moustiques comme agents transmetteurs.

Mais hors d'Europe, comment étaient perçues l'origine et la propagation de ces « fièvres »?

Selon Mario Maffi [21], des Massaïs des Monts Usambara (Tanzanie) et peut-être les Galla (Oromo) d’Éthiopie et de Somalie, auraient établi entre paludisme et moustiques un lien concrétisé par la désignation par le même mot de la fièvre et de l'insecte: « Mbu » en Tanzanie, « Canda-dilmaio (ou dilmagno) » en Somalie^6^6Ugo Ferrandi, lors de ses explorations de l’Éthiopie et de la Somalie dans les années 1880-90, n'admettait pas cette croyance d'un lien établi entre paludisme et moustiques. Son scepticisme venait du fait que, dans son esprit, les indigènes « ne savent pas encore que le paludisme est dû à des miasmes »!. Tout comme d'autres populations africaines ont lié glossines et nagana (utilisant un même nom pour désigner les glossines et la maladie en langue bantoue).

Autrement dit, alors qu'en Europe on incriminait en premier lieu le caractère « malsain » des marais, les eaux stagnantes, la vase, les émanations et l'air putride qui s'en dégageaient, parfois des « animalcules » sans évoquer les moustiques, en Afrique, des populations accusaient clairement les moustiques.

D'où provient cette différence d'approche? Pour Mario Coluzzi [8], l'explication est à rechercher dans la bio-écologie des différents anophèles. En Europe, les vecteurs sont des *Anopheles* du complexe *Maculipennis* (en particulier *An. labranchiae, An. maculipennis, An. atroparvus, An. sacharovi, An. messeae*) dont les gîtes larvaires sont constitués par des collections d'eau stagnante, souvent permanentes, situées en zone basse, marécageuse et produisant de très nombreux moustiques. En Afrique, en revanche, le lien entre paludisme et anophèles serait plus facile à reconnaître car les vecteurs majeurs, des *Anopheles* du complexe *Gambiae* (en particulier *An. arabiensis* et *An. gambiae* s.s.), *An. funestus* ou *An. nili*, ont des larves se développant dans différents types de gîtes (pas toujours des marécages mais plutôt des mares résiduelles, temporaires) et la présence d'anophèles anthropophiles est constante dans tous les environnements, d'où une perception populaire davantage centrée sur une association avec les moustiques.

Mais le véritable mode de transmission de la maladie demeurait un mystère.

Laveran n'aimait pas le terme « malaria » adopté par les auteurs français, pas plus que « fièvre malarique ». Dans ses livres, il utilise plutôt « paludisme » ou « fièvre palustre ». Néanmoins, cela ne lempêcha pas de donner le nom de *malariae* à son hématozoaire.

Ce n'est qu’à la fin du xix^e^ siècle que les choses se précisent enfin. Que s'est-il passé? Reportons-nous par la pensée à cette époque héroïque des vingt dernières années de ce siècle.

Le concept de transmission de maladies infectieuses par des insectes faisait son chemin et, progressivement, pour ce qui est du paludisme, la « mosquito theory » comptait de plus en plus d'adeptes. Aux États-Unis d'une part, où, dès 1807, John Crawford, de Baltimore, avait suggéré la possibilité d'une transmission de l'agent du paludisme (alors inconnu) par des moustiques, suivi en cela par Josiah Nott de Mobile (Alabama) en 1848 et par Albert Freeman King, gynécologue, qui, en 1882, ignorant encore la découverte de Laveran, aurait préconisé d'entourer Washington d’écrans de fils tressés, une sorte de moustiquaire géante (il ne fut pas suivi dans ce projet !).

## Transmission Des Agents Du Paludisme: Les Hypothèses À La Suite Des Travaux De Manson

Il n'est pas douteux que l'observation, par Patrick Manson, réalisée à Amoy (Xiamen) en Chine en 1877, de l'implication des moustiques dans le cycle naturel de la filaire de Bancroft eut un impact décisif en attirant vers ces insectes l'attention des malariologistes. Toutefois, à l’époque de sa découverte, Manson ne croyait pas que le moustique fût directement responsable de la transmission de la filaire; il pensait que l'insecte infecté, se noyant dans une collection d'eau, libérait les larves du parasite qui étaient ensuite ingérées avec l'eau en question. Il faut dire que Manson lui-même, qui n’était pas entomologiste, ne savait pas identifier les différentes espèces; il pensait que la longévité des moustiques était très courte, qu'ils ne prenaient qu'un seul repas sanguin et que, de ce fait, ils ne pouvaient transmettre par leur piqûre. Pour lui, les moustiques mouraient aussitôt après avoir pondu une unique fois. Néanmoins, il s'agit là d'une étape essentielle dans la progression des idées dans ce domaine. L'entomologie faisait irruption dans le champ de la médecine. Désormais, l'idée était dans l'air et progressivement, la « mosquito theory » faisait son chemin.

Robert Koch en Inde (1883-84) a également soupçonné le rôle des moustiques. Toutefois, il suggérait encore, à Berlin en 1898, que les moustiques pouvaient pondre leurs œufs sur le corps de l'homme et que le parasite pouvait alors s'en échapper pour passer dans le sang.

L'idée de la transmission par moustiques était soutenue en Allemagne par Carl Flügge en 1889. Et en 1892 (soit 5 ans avant Ross), Richard Friedrich Pfeiffer, influencé par Koch, suggérait, par analogie avec les coccidies, que l'hématozoaire de Laveran devait avoir un cycle se déroulant en partie chez des insectes et qu'il pourrait être transmis par des insectes hématophages. Cependant, il y avait autant de détracteurs que de soutiens à cette théorie.

L'idée d'une transmission par arthropode hématophage s’était par ailleurs trouvée renforcée par les écrits de Louis-Daniel Beauperthuy à Caracas en 1854-56, puis par les travaux de Finlay à La Havane en 1881 sur la propagation du virus de la fièvre jaune. Les découvertes de cet ordre s'enchaînèrent ensuite rapidement, que ce soit pour la peste, les trypanosomoses, les babésioses, le typhus, la maladie de Chagas, les fièvres récurrentes (borrélioses), etc.

Quelle était la position d'Alphonse Laveran dans le contexte de l'histoire des idées sur la transmission du paludisme, dans cette période extrêmement riche en découvertes, entre 1880 et 1910?

Dès 1884, dans son *Traité des fièvres palustres*, Laveran avait déjà émis l'idée que des moustiques pourraient bien se trouver impliqués dans la transmission des hématozoaires. Comme le souligne Laverdant, cette hypothèse, chez Laveran, résultait de l'observation et de la réflexion d'un hygiéniste averti [19]. Le fait que l'agent du paludisme soit un parasite, et non une bactérie, incita aussitôt Laveran à considérer la probabilité d'un cycle de développement dans le milieu naturel: « Les insuccès des essais de culture m'ont conduit à croire que le microbe vivait dans le milieu extérieur à l’état de parasite. » Il estimait que les « corps flagellés » représentaient des formes d’évolution du parasite et que celui-ci « devait parcourir une phase de son existence dans un hôte animal et, vraisemblablement dans les moustiques qui infectent les régions palustres. […] Je suis arrivé à la conviction que le microbe se trouvait en dehors du corps de l'homme, à l’état parasitaire et très probablement à l’état de parasite des moustiques [22]. »

Manson, lui aussi, observant l'exflagellation des *Plasmodium*, avait rapidement compris qu'il s'agissait d'un stade de développement du parasite destiné à se développer ultérieurement dans l'organisme d'un insecte hématophage et pensa à l’éventualité d'une transmission par moustiques. Il a cherché à vérifier cette hypothèse en tentant d'abord de se rendre en Guyane britannique (mais il n'a pas obtenu le financement nécessaire), puis de faire des expériences de transmission dans une serre du Jardin botanique de Kew; en vain.

Comme Manson à propos de la filariose, beaucoup estimaient que la contamination par l'hématozoaire devait survenir lorsque l'on buvait de l'eau contenant des parasites issus de moustiques qui s'y étaient noyés (la transmission de la filaire par la piqûre du moustique ne sera démontrée par Bancroft qu'en 1899 en Australie puis par Low en 1900 en Angleterre). Il faut dire qu’à cette époque, on ne savait pas grand-chose sur la vie des moustiques.

En 1894, au Congrès d'Hygiène de Budapest, Laveran, qui suspecte toujours fortement les moustiques, reçoit l'adhésion de Manson qui pense encore que les parasites seraient libérés dans l'eau. Dans son *Traité des fièvres palustres*, Laveran, évoquant la théorie de Manson de la transmission par l'eau, écrit: « Le moustique a-t-il un rôle similaire dans la pathogenèse du paludisme? Cela n'est pas impossible et c'est un fait notable que les moustiques abondent dans toutes les zones impaludées. »

Mais, comme le dit Laveran lui-même, « l'opinion que je défendais était considérée, jusqu'en 1895, par la plupart des observateurs, comme très peu vraisemblable [22] ». Ainsi, en 1892, deux auteurs italiens, Grassi et Feletti, qui sont devenus par la suite de grands partisans de l'infection par les moustiques, écrivaient: « Laveran suppose que les moustiques sont les hôtes intermédiaires du parasite palustre. Nous objectons que les moustiques ne s'attaquent pas aux oiseaux [.] et, en outre, qu'il y a beaucoup de localités salubres où abondent les moustiques. En dehors de ces objections, il a été constaté par Calandruccio que les parasites du paludisme meurent dans l'intestin des moustiques sans développement ultérieur. L'opinion de Laveran reste donc sans fondement et l'hypothèse émise par nous que les parasites existent dans le milieu extérieur sous forme d'amibes se confirme. »

En France également, François-Henri Hallopeau « trouve invraisemblable l'intervention des moustiques dans la propagation du paludisme ».

## Transmission Des Agents Du Paludisme: Les Travaux De Ross

Manifestement, près de 15 ans après la mise en évidence de l'hématozoaire, la preuve scientifique s'avérait difficile à établir.

C'est alors qu'intervient Ronald Ross. Lors de sa première affectation comme médecin militaire à Madras, il y avait découvert les moustiques: son bungalow en était plein et ces insectes l'agaçaient prodigieusement.

En 1894, à Londres, Patrick Manson persuada Ronald Ross, qui repartait aux Indes, de poursuivre les travaux et de démontrer que l'agent du paludisme était bien transmis par des moustiques, même si, au début, Ross n'avait pas réussi à voir les *Plasmodium* dans le sang des malades et ne croyait pas à la découverte de Laveran.

Les nombreuses difficultés, tant techniques qu'administratives, rencontrées sur place par Ronald Ross ont été maintes fois relatées [12] et nous n'y reviendrons pas ici (voir note complémentaire en fin d'article). On retiendra surtout qu'il montra que l'exflagellation intervenait normalement dans l'estomac des moustiques, qu'il découvrit un autre stade du parasite – l'oocyste, et que le développement du parasite se réalisait chez des moustiques. Suivant les idées de Manson, il tenta en 1895 d'infecter des hommes par ingestion d'eau « contaminée » par des moustiques morts, puis il chercha à montrer une contamination par les gouttelettes émises par les moustiques sur la peau, toujours sans succès. Il pensa alors à une infection par la piqûre du moustique. Une expérience fut réalisée sur un volontaire piqué par 5 moustiques infectés deux jours plus tôt par repas sanguin: résultat négatif; il n'avait pas attendu assez longtemps pour que s'accomplisse le développement du parasite (erreur que Finlay avait déjà faite à Cuba avec le virus amaril). De plus, beaucoup des expériences entreprises par Ross restèrent négatives parce qu'il se servait le plus souvent de moustiques les plus communs, des *Culex* et non des *Anopheles^7^7Le genre *Anopheles* avait été décrit par Meigen en 1818 mais on n'en connaissait, en cette fin du xix^e^ siècle, qu'une vingtaine d'espèces; elles sont aujourd'hui environ 480 dont une soixantaine peuvent transmettre les agents des paludismes humains..* Lors de ses travaux sur le paludisme des oiseaux, il put enfin observer que les oocystes donnaient des formes parasitaires qui gagnaient les glandes salivaires; la transmission par piqûre devenait alors très vraisemblable.

Il faut reconnaître que même si, à la suite des travaux de Manson, la transmission de l'agent du paludisme par moustique a d'abord été envisagée puis finalement acceptée, personne n'imaginait l'existence d'un cycle complexe du parasite dans l'organisme du moustique.

Pour l'ensemble de ses travaux sur la transmission des hématozoaires du paludisme, Ronald Ross se verra attribuer le prix Nobel en 1902.

Une part du mérite doit cependant revenir à Patrick Manson qui a constamment guidé et conseillé Ross dans ses travaux [6]. À ce propos, Broquet écrit, en 1929: « L'on ne peut vraiment séparer les noms de Manson et de Ross; ni l'un ni l'autre ne l'eussent permis. De Londres, le premier conseillait, encourageait, contrôlait; dans l'Inde, le second méditait, parfois rectifiait les conseils du maître, puis cherchait et découvrait [25]. »

Plus tard, Ross lui-même écrit très honnêtement: « Il est remarquable que le Dr. Laveran n'ait pas seulement été le premier à observer l'agent du paludisme mais aussi le premier à indiquer son mode de développement en dehors de l'organisme humain [24]. »

## Transmission Des Agents Du Paludisme: Les Travaux Des Chercheurs Italiens. Querelles Et Collaborations, Scepticisme Persistant

Mais l'histoire ne s'arrête pas aux observations de Ronald Ross, aussi fondamentales fussent-elles.

En Italie, on avait abandonné l'idée des eaux putrides des marais. En 1896 déjà, Amico Bignami se dit convaincu, dans une lettre au *Lancet*, de la transmission par moustiques, théorie soutenue en 1898 par Grassi et Bastianelli ainsi que par Robert Koch.

Évidemment, la découverte de Ross est aussitôt connue en Italie et, cette même année 1898, Bastianelli décrit les stades de développement du parasite humain chez « *An. claviger* » (probablement *An. labranchiae*). En quelques mois, Grassi, Bastianelli et Dionisi ont décrit les cycles de *P. falciparum* et de *P. vivax* chez l'anophèle (novembre 1898). L'année suivante, ils montrent aussi que seuls les anophèles transmettent, qu'une seule piqûre suffit et que, chez les moustiques, le parasite n'est pas transmis à la descendance^8^8Certains pensaient qu'il pourrait exister une transmission verticale du parasite chez les moustiques [33].. Les chercheurs italiens connaissaient bien, et depuis longtemps, la biologie des moustiques, en particulier des *Anopheles* (travaux de Ficalbi, de Fermi, etc.) [27]. Mais Grassi pensait alors que toutes les espèces italiennes *dAnopheles* transmettent, et même tous les *Anopheles* du monde [11]. En 1900, Laveran semble être de cet avis (cf. *La Presse Médicale* du 24 octobre 1900).

Une expérience est alors entreprise à l'automne 1898: Bignami récolte des « *An. claviger* » dans une zone impaludée et, à l'hôpital Santo Spirito de Rome, les lâche dans la chambre d'un malade volontaire non impaludé, hospitalisé depuis 6 ans, qui contracte un paludisme sévère avec récurrences. L'expérience est répétée en décembre 1898 et janvier 1899; les résultats en sont publiés en 1900 [7]. Cette observation fait l'objet d'une communication, le 28 novembre 1898, à l'Academia dei Lincei. Nous sommes là 18 années après la découverte de Laveran.

Une critique toutefois se fait jour à propos de ces expériences réalisées à l'hôpital Santo Spirito: les volontaires piqués par ces moustiques sont italiens, et donc susceptibles d'avoir été contaminés au préalable. Dès lors, après une visite de Manson en Italie, de nouvelles expériences s'imposent; à son instigation, elles sont mises sur pied avec la collaboration de chercheurs britanniques.

En premier lieu, une mission fut envoyée d'Angleterre en Italie en 1900. Elle était composée de G. Carmichael Low, Luigi Sambon (tous deux de la London School of Tropical Medicine) et de A. Terzi (dessinateur du British Museum, d'origine italienne). Tous trois séjournèrent, durant les trois mois de la saison de transmission (juillet-octobre), près d'Ostie, dans une zone très fortement impaludée de la Campagne romaine, vivant normalement le jour mais s'enfermant, dès la nuit tombée, dans une maison aux fenêtres ouvertes mais munies de moustiquaires (cette maison avait été fabriquée en Angleterre et assemblée sur place, à Castel Fusano, dans une propriété appartenant au roi d'Italie Umberto I^er^)^9^9Umberto I^er^ fut d'ailleurs assassiné à Monza le 29 juillet 1900, durant la présence à Ostie des chercheurs britanniques.; ainsi exposés « à l'influence pestilentielle de cette vallée de l'ombre de la mort », ils sont demeurés parfaitement indemnes [1].

Une autre expérience fut alors décidée conjointement par les Italiens et les Anglais. Il s'agissait d'envoyer en Angleterre des anophèles préalablement gorgés sur un malade à l'hôpital Santo Spirito, puis de leur faire prendre un repas sanguin sur des personnes n'ayant jamais résidé en pays d'endémie palustre. Aussitôt dit, aussitôt fait. Bignami et Bastianelli envoyèrent des *An. labranchiae* infectés; à Londres, les insectes furent gorgés sur le fils de Patrick Manson (Patrick Thorburn Manson) et sur George Warren, assistant à la London School of Tropical Medicine. Tous deux présentèrent 14 jours plus tard les premiers signes d'un paludisme à *P. vivax*.

Considérée comme décisive, cette dernière expérience marque la fin des études sur la « mosquito theory » de Manson. Nous sommes dès lors entrés dans le domaine de l’épidémiologie biologique. Les entomologistes allaient devoir se lancer dans l’étude détaillée de la biologie des anophèles, les parasitologues dans celle des *Plasmodium*. Et les deux disciplines allaient être conjointement sollicitées pour l’étude des systèmes anophèles-plasmodies.

Il est intéressant de considérer, comme le fait Harrison [16], les différences dans l'approche du problème entre les équipes: « Bignami s'intéressait à la façon dont le parasite passait de l'environnement au sang de l'Homme alors que Manson se focalisait sur la façon dont le parasite sort de l'organisme pour rejoindre l'environnement. L'un et l'autre ont vu deux moitiés du cycle. Pour Bignami, le moustique était une seringue inoculatrice; pour Manson, il était une pipette et un moyen de transport. Aucun d'eux ne s'est aperçu que la trompe pouvait être un système à deux sens, pour l'entrée et la sortie. »

Il n'en demeure pas moins que la rivalité entre Grassi et ses collaborateurs d'une part, et Ross soutenu par de nombreux collègues britanniques d'autre part, engendra une violente polémique. Cette querelle pour la priorité de la découverte fut parfois très vive, agrémentée d'insultes orales et écrites, les Italiens étant qualifiés de « brigands », les accusations de faux et d'escroquerie fusant de part et d'autre [15, 24]^10^10Il faut rappeler ici que Ronald Ross était d'un caractère ombrageux, volontiers jaloux et rancunier, même vis-à-vis de Manson qui l'avait pourtant initié, guidé et soutenu. Ross était aigri, pensait sans cesse être dévalué et ceci transparaît bien dans ses *Mémoires* autobiographiques publiés en 1923, écrits dans un style souvent polémique; on imagine volontiers qu'il n'attirait pas spontanément la sympathie de tous. Il se plaignait sans cesse: de ne pas avoir de chaire assez tôt, de ne pas avoir un accès facile aux malades « tropicaux », de n’être pas suffisamment payé ou, après avoir quitté la Liverpool School of Tropical Medicine, de ne pas toucher une pension de la part de cet organisme.. Bien entendu, la polémique prit encore plus d'ampleur en 1902, à l'annonce de l'attribution du Prix Nobel de médecine au seul Ronald Ross, au grand mécontentement de Grassi. Mais, comme le suggère Bynum [5], si, comme ce sera le cas plus tard, le prix avait pu être décerné à plusieurs personnes, il aurait sans doute été partagé avec Grassi ou Manson ou encore Laveran (ce dernier devait recevoir le prix en 1907).

Ceci dit, le rôle des anophèles dans la transmission du paludisme humain ne fut pas d'emblée accepté par tous, loin de là. Des faits pouvaient en effet plaider contre cette hypothèse, en particulier: 1) il existe beaucoup de zones d'anophélisme sans paludisme et ce n'est pas une question de climat (le paludisme pouvait s'observer jusqu’à Arkhangelsk); 2) beaucoup d'anophèles ne transmettent pas les *Plasmodium* humains; 3) des *Plasmodium* d'animaux sont transmis par d'autres vecteurs que des anophèles, etc.

Relisons les journaux de l’époque et reprenons quelques-uns des arguments avancés par les opposants à cette théorie.

Beaucoup de ces opposants font état de « fièvres » survenant en l'absence de ces moustiques, chose difficile à démontrer, ou à la survenue d’épisodes fébriles dans la population mais sans diagnostic précis.

Le Dr Devaux, en 1903, décrit une épidémie de malaria sans moustiques au Tonkin: « pendant cette épidémie de malaria si violente, si funeste, où les accès pernicieux surgirent de tous côtés, les moustiques n'ont pas été plus communs que d'habitude; et ils sont rares dans cette région, si rares que l'Européen y dort sans moustiquaire. […] Cela ne veut pas dire que l'anophèle ne puisse pas être un agent propagateur des plus dangereux de la malaria, mais il n'est certainement pas le seul [9]. »

En 1910, le Dr Grandmaire [13] estime que la piqûre du moustique n'est pas le facteur primordial de l'infection palustre et qu'il convient de rendre aux causes inhérentes au milieu extérieur la place importante qu'elles occupaient autrefois.

Pour expliquer la survenue d’épidémies, la plupart évoquaient le rôle du sol, des grands remuements de terre ou de vase (curage du Penque à Bordeaux en 1805, creusement du canal Saint-Martin à Paris en 1811, établissement des fortifications autour de Paris en 1840, etc.): « On est forcément amené à considérer comme négligeable ou même comme à peu près nul le rôle qui a pu être joué par les moustiques en pareilles circonstances, on n'envisage plus le fait primordial dont l'importance domine tout: le grand remuement de terre ou de vase qui dégage des gaz émanés de matières organiques en putréfaction [20]. » Même en acceptant le principe de la transmission par anophèle, Le Ray pose une question complémentaire: s'agit-il du seul mécanisme de transmission de l'hématozoaire? Il écrit, à propos de la survenue de cas de paludisme à la suite de grands travaux de terrassement: « La coïncidence étroite du développement de l’épidémie avec l'exécution des travaux, la répétition des mêmes phénomènes morbides chaque fois que des détritus sont ramenés à l'air libre ne laissent dans l'esprit place à aucun doute: la maladie a jailli du sol [20]. »

Le rôle du sol restera longtemps très ancré dans les esprits, avec des arguments souvent de très peu de valeur, comme ceux avancés par Braddock en 1907 [3] dans les forêts de Thaïlande: pour lui, dans une expédition au cœur de la jungle, quelle que soit la saison, les hommes à pied contractent les fièvres palustres, les hommes à cheval ne les contractent que rarement, ceux qui sont à dos d’éléphant pratiquement jamais; ceux qui sont en hauteur au-dessus du sol ne sont presque pas exposés aux miasmes provenant du sol et des végétaux pourrissants. Certains sont longtemps restés fidèles aux idées antérieures. Encore dans les années 1930, par exemple, Wolter, de Hambourg, qui avait étudié le paludisme en Russie, ou l'Italien Bellincioni en 1934, estimaient qu'un facteur environnemental du sol ou le niveau des nappes phréatiques étaient impliqués dans l'existence ou non du paludisme dans une région.

Le scepticisme persistait donc. Ross lui-même estimait que le monde avait besoin de 10 ans pour assimiler une idée nouvelle, fusse-t-elle importante et simple. Il se trompait: il faut, je crois, davantage de temps encore.

Il faut également reconnaître que la situation est rendue complexe par le fait que certaines espèces d'anophèles peuvent être vectrices ici et non là. Le problème est lié à la compétence vectorielle des anophèles vis-à-vis des différents *Plasmodium* et à l'existence de complexes d'espèces jumelles.

Ainsi, Le Ray écrit: « Au Tonkin, par exemple, il est de notoriété publique que le paludisme déserte la plaine où foisonnent les anophèles et sévit surtout au sommet des mamelons où les moustiques sont tellement rares que l'on ne ressent presque jamais leurs piqûres, bien que la plupart du temps on ne fasse point usage de moustiquaire [20]. » On sait très bien aujourd'hui qu'avant que soient entreprises des opérations de déforestation et de lutte anti-anophélienne, sévissaient, dans les collines forestières de cette région, des membres des complexes *An. minimus* et *An. dirus*, vecteurs très efficaces, alors que les plaines basses n'hébergent que des anophèles, certes abondants, mais à faible compétence vectorielle.

Un tel phénomène avait déjà été remarqué par les Italiens avec *An. maculipennis* dont Falleroni a décrit en 1926 deux formes: *An. labranchiae* et *An. messeae*; d'autres formes de ce complexe ont été décrites par la suite, en Italie comme en Allemagne et aux Pays-Bas, dont certaines ne sont pas anthropophiles.

N. H. Swellengrebel, dans l'article « Le médecin et l'entomologiste » de 1935 [31], rappelle aussi qu'au début du xx^e^ siècle, au Bengale, Stephens et Christophers, voulant étudier la corrélation entre la distribution géographique du paludisme et celle des anophèles, se trouvèrent étonnés car leurs résultats démontrèrent clairement que cette corrélation n'existait pas, sinon dans le sens opposé à leurs attentes: anophèles rares dans les régions impaludées, anophélisme le plus dense dans les régions indemnes. L'analyse systématique de la faune anophélienne du Bengale montrera plus tard qu'elle était composée d'espèces différentes, chacune avec ses caractères bioécologiques propres, notamment pour ce qui est de leur compétence vectorielle. Ceci en surprit beaucoup qui n'acceptèrent pas cette conclusion.

Pourtant, dès 1920, Émile Roubaud avait attiré l'attention sur ce phénomène et avancé le concept d’« anophélisme sans paludisme ».

## Les Relations *Plasmodium-Anopheles* Dans L'esprit De Laveran

Force est de reconnaître que les Français n'ont pas joué de rôle réellement déterminant dans cette affaire.

**Laveran** était un protozoologiste, excellent au demeurant. Le fait de reconnaître un *Plasmodium* à l'examen direct du sang, sans coloration (les premiers procédés de coloration des globules sanguins n'ont été mis au point qu'en 1891 par Romanovsky à Saint-Pétersbourg) et à l'aide d'un microscope des plus modestes, dépourvu d'objectif à immersion, montre ses exceptionnelles qualités d'observateur.

Mais il n’était pas entomologiste, comme le furent un peu plus tard Edmond Sergent et Émile Roubaud. Il connaissait peu les moustiques tant sur le plan de la systématique que sur ceux de la biologie et de l’écologie. Roubaud écrit: « Laveran s'aventurait ici dans un domaine qui n’était pas le sien, bien qu'il l'intéressât fortement. Il y avait tout à apprendre et, scrupuleusement, enregistrait tout ce qu'il observait, marquant ainsi son perpétuel souci de ne méconnaître aucun détail [26]. » Laveran travaille sur des moustiques qui lui sont envoyés de partout. Cependant, sur ses quelque 600 publications, il ne publie en tout et pour tout qu'une quinzaine de papiers sur la systématique des Culicides, portant notamment sur des espèces d'Indochine, de Nouvelle-Calédonie, des Nouvelles-Hébrides (Vanuatu), de Djibouti ou de Madagascar. On notera que c'est lui qui décrit une espèce nouvelle, *Anopheles farauti*, qui se révélera être le grand vecteur du paludisme en Mélanésie. Dans ces articles, Laveran décrit en effet quelques espèces qu'il estime nouvelles mais n'en donne généralement que des descriptions succinctes et, faute de collection de référence, ces espèces se trouveront souvent mises en synonymie par la suite. En fait, Laveran établit surtout ses listes d'espèces culicidiennes qu'il utilise pour montrer la coïncidence entre présence d'anophèles et incidence du paludisme et donc conforter ainsi le rôle des anophèles. Il souligne l'absence d'anophèles en Nouvelle-Calédonie et à Tahiti, pays non impaludés, alors qu'ils abondent aux Nouvelles – Hébrides (Vanuatu), pays d'endémie palustre.

Laveran a évidemment beaucoup réfléchi au problème de la transmission des *Plasmodium* mais, en pratique, il a peu travaillé lui-même sur la « mosquito theory ». À la suite de Ross et de Grassi, Laveran figure parmi les premiers défenseurs de la théorie anophélienne. De 1900 à 1903, il prend une part active dans l’établissement des rapports qui peuvent exister entre les anophèles et le paludisme; il voyage en Corse, en Camargue. Dès 1901, dans un rapport à l'Académie de médecine, il demande la création d'une Société pour l'assainissement de la Corse et trace le programme des mesures à employer. Cette Société est créée l'année suivante à Bastia, sous le nom de « Ligue corse contre le paludisme ». En Algérie, une autre ligue contre le paludisme est fondée en 1903 à Alger [32]. Laveran assure la présidence de ces deux institutions.

Laveran s'intéresse de près aux conditions environnementales qui favorisent la propagation du paludisme. « Si le marais constitue le milieu le plus favorable au développement du paludisme, il ne suffit pas par lui-même à produire l'endémie, même dans les conditions de climat les plus favorables [18]. » Et, bien sûr, il réfléchit à l'importance de la répartition saisonnière des pluies, des inondations, des cyclones, etc., mais aussi des travaux de terrassement, des grands remuements de sol, du creusement de canaux, etc. qui entraînent la formation de mares d'eau stagnante après la pluie, qui peuvent causer des épidémies de paludisme (il cite en exemple le canal Saint-Martin creusé en 1811, les travaux réalisés en Algérie et ceux de la ligne de chemin de fer à Madagascar), ou encore sur le rôle de l'altitude.

À propos du danger présenté par les rizières, Laveran [18] cite Vivarelli: « Les rizières produisent du riz et des fièvres graves; la récolte du riz peut manquer, celle des fièvres est toujours abondante. » Ce danger est souligné par les observations faites au Tonkin, à Madagascar et en Camargue.

De plus, dans son *Traité du paludisme* de 1907 [18], Laveran précise: « Il faut que la température extérieure s’élève suffisamment, au moins pendant une partie de l'année, et qu'il y ait de l'eau stagnante. […] Si la température est trop basse, les larves de moustiques ne peuvent pas se développer et surtout *Haemamoeba malariae* [nom désignant alors le *Plasmodium*] ne peut pas accomplir son évolution dans les Anophèles. » Il avait donc une vue assez claire des facteurs qui conditionnent l'incubation extrinsèque des *Plasmodium* chez les vecteurs.

## Transmission Des Agents Du Paludisme: Les Conséquences Pratiques Pour La Lutte Antipaludique

Au total, avec 1880, l'année 1898 fut l'une des plus significatives, une *annus mirabilis* selon Bruce-Chwatt [4], dans l'histoire de la malariologie et, plus largement, l'histoire de la médecine tropicale. Ross, à Calcutta, a montré le cycle complet du *Plasmodium* sur des oiseaux tandis que les Italiens Grassi, Bignami et Bastianelli et leurs collaborateurs ont conduit leurs expériences sur la transmission des *Plasmodium* humains, décrivant le cycle complet chez le moustique et furent les premiers à obtenir la preuve scientifique de la relation spécifique entre les anophèles et le paludisme humain^11^11Rappelons ici que ce nest quen 1948 que les phases pré-érythrocytaires du cycle des Plasmodies furent décrites par Shortt, Garnham, Covell et Shute pour *P. vivax*, et l'année suivante pour *P. falciparum* par Shortt, Fairley, Covell, Shute et Garnham.. Dans ce contexte de nationalisme exacerbé, Grassi annonça triomphalement: « La clé était enfin trouvée, la porte s'est ouverte et la lumière a brillé depuis l'Italie. »

Optimiste, Ross écrivait peu après sa découverte: « Dans quelques mois, peut-être une année ou deux, la maladie mortelle devrait commencer à être contrôlée, à diminuer dans des zones favorables, puis, lentement, la maladie ubiquitaire va disparaître de la surface des pays civilisés, pas seulement ici ou là, mais pratiquement partout dans l'Empire britannique. Puis en Amérique, en Chine et en Europe. » La réalité, on le sait, fut bien différente: même si l'incidence de la maladie a fortement baissé, l'endémie palustre sévit toujours dans nombre de pays, en particulier en Afrique. De son côté, Laveran écrit dans son *Traité du Paludisme* [18]: « On ne doit plus se résigner nulle part à l'endémie palustre comme à un mal inévitable, inséparable des conditions climatiques ou telluriques de certains pays; la fièvre palustre doit être classée désormais parmi les maladies évitables. » On peut évoquer ici les travaux sur la lutte antipaludique menés par les frères Sergent en Algérie, bien décrits dans leur publication *Vingt-cinq années d’étude et de prophylaxie du paludisme en Algérie* [29], ou encore l'assèchement des marais Pontins aux environs de Rome et leur conversion en terres agricoles qui furent entrepris avec succès dans les années 1930, après les importants travaux d'assainissement menés dans la Maremme à partir des années 1820. L’élucidation du mécanisme de la transmission du paludisme devait en effet ouvrir une ère d'espoir. « Désormais, il était possible non seulement de reconnaître avec certitude la maladie et de la guérir, mais encore d'en expliquer l’épidémiologie et surtout de s'en préserver: l’ère de la prophylaxie du paludisme, basée sur la défense contre les moustiques, commençait [2]. » Il devint possible, durant la première moitié du xx^e^ siècle, d’élaborer des techniques de prévention du paludisme par la lutte anti-anophélienne: drainage et assèchement des marais avec mise en culture des terres, pétrolage, épandage de vert de Paris (à partir de 1916), recours au pyrèthre (à partir de 1918), protection personnelle par moustiquaire, en parallèle avec la chimioprophylaxie par la quinine et ses dérivés et, par la suite, avec le recours aux insecticides rémanents.

## Qui Est Ronald Ross?

Ronald Ross (1857-1932) est né aux Indes, dans une de ces stations climatiques d'altitude, aux flancs de l'Himalaya, qu'affectionnaient tant les Anglais. Son père, Campbell Ross, alors capitaine dans un régiment de Gurkhas, y était en poste; il fit campagne sur la frontière du Nord-Ouest avant d’être affecté en 1864 à Bénarès; il devint par la suite le général Sir Campbell Ross.

Le jeune Ronald, aîné d'une fratrie de 10 enfants, fut envoyé en Angleterre à l’âge de 8 ans. Adolescent, il aspirait plutôt à l'art, la musique, la poésie… Pas très sérieux, tout cela, aux yeux du général, qui décida que son fils servirait, comme lui, dans l'Armée des Indes ! Après avoir poursuivi en Angleterre ses études secondaires, puis médicales (à vrai dire pas particulièrement brillantes, au point de rater l'examen de la Society of Apothecaries, ce qui empêcha son recrutement direct par l'Indian Medical Service, si bien qu'il dut passer un an comme chirurgien navigant avant de tenter à nouveau sa chance), il ne put décrocher qu'une affectation considérée comme la moins prestigieuse: Madras.

« C'est là qu'il découvrit les moustiques. Son bungalow en était plein et les insectes l'agaçaient prodigieusement » [23]. Il y resta de 1881 à 1888, passant le plus clair de son temps, nous dit W. F. Bynum, à chasser, pêcher, jouer au golf et écrire poèmes et pièces de théâtre. En 1888, en permission en Angleterre, il y compléta sa formation en microbiologie. Et c'est muni d'une épouse et d'un microscope, qu'il retourna aux Indes pour un second séjour (1889-1894), principalement à Bangalore. C'est lors d'un autre retour à Londres que, pour la première fois, Ross observa des *Plasmodium* sur des lames de sang que lui montra Manson; nous sommes alors en 1894. Dès lors, affecté comme médecin militaire à l'hôpital de Secunderabad à partir du 13 mai 1895, il n'eut de cesse de démontrer la « mosquito theory » de Manson.

Ronald Ross entreprit donc des travaux sur la transmission du paludisme. Il nourrissait des moustiques d'espèces communes sur des porteurs de gamétocytes, puis les disséquait, espérant observer des stades plus avancés des parasites. En vain. Les déceptions succédèrent aux déceptions. Les échecs étaient la règle car il ne travaillait pas sur les bonnes espèces; il faut dire que Ross ne connaissait pratiquement rien aux moustiques. Et ce durant deux ans et demi, dans un environnement dominé par l'indifférence et le scepticisme général, jusqu’à une nouvelle série d'essais pratiqués en 1897 sur une autre espèce culicidienne, en réalité un anophèle. Les expériences furent donc très nombreuses et c'est sans doute quelque peu arbitrairement que, parmi d'autres, la date du 20 août 1897 fut retenue.

L'expérience décisive débuta le 16 août 1897. Ce jour-là, Ross fit gorger des moustiques, très probablement des *An. stephensi*, sur un patient infecté par *P.falciparum*, du nom de Hussein Khan. Tous les jours suivants, il disséqua quelques-uns de ces moustiques. Le 19 août, il remarqua l'existence de « cellules vacuolées particulières » sur l'estomac des insectes et, le lendemain, le 20 août (le fameux « mosquito day »), il trouva sur la paroi de l'estomac une cellule de ce type contenant le pigment considéré comme caractéristique depuis les observations de Heinrich Meckel von Hemsbach (en 1847, Meckel fut peut-être le premier d'une assez longue suite de chercheurs à avoir vu le parasite sans réaliser qu'il s'agissait de l'agent du paludisme) et celles de Rudolf Virchow en 1848. Ross en conclut qu'il s'agissait d'un stade particulier de l'hématozoaire du paludisme chez le moustique (ce que nous appelons aujourd'hui les oocystes). Le mois suivant, il observa plusieurs oocystes sur l'estomac d'un autre moustique.

Malheureusement, dès la fin septembre, les circonstances l'obligèrent à interrompre ses recherches à la grande déception de Manson qui voyait la rapide progression des travaux italiens: il était envoyé à Bombay. L'année suivante, en janvier 1898, Ross fut affecté à Calcutta; là, il tenta de reprendre ce travail mais les nombreuses difficultés techniques (peu de moustiques, peu de malades et encore moins de volontaires) finirent par le dissuader de poursuivre ses travaux sur le paludisme humain et l'amenèrent à se tourner vers des plasmodies d'oiseaux (*P. relictum*) transmis par *Culex* (on ne connaissait pas encore les *Plasmodium* des rongeurs). Ici encore, les expériences du début furent infructueuses mais, au bout de quelque temps, il réussit à nouveau à obtenir des corps pigmentés chez certains de ses moustiques nourris sur oiseaux parasités par ce que l'on appelait alors des *Proteosoma.* Ainsi se trouva résolu le problème scientifique de base. Cette découverte fut présentée par Manson à la British Medical Association d'Edinburgh le 24 juillet 1898 (et publiée le 24 septembre 1898).

Temporairement affecté à Darjeeling pour travailler sur le kala-azar et sur la peste, Ross dut encore interrompre son travail. Mais, revenu à Calcutta, il reprit ses expériences. Il observa que la taille des oocystes augmentait avec le temps, en perdant progressivement leur pigment, que la rapidité de cette croissance dépendait de la température: leur évolution durait de 6 jours en saison chaude à 2 semaines ou plus en saison fraîche. Il se rendit compte enfin que les oocystes donnaient naissance soit à des éléments allongés, très fins, de 12 à 16 μm de long (ce sont évidemment les sporozoïtes), soit à des éléments arrondis et sombres, qu'il appela les « black spores ».

Il s'interrogea sur la signification de ces deux types d’éléments qu'il retrouvait chez ses insectes. Le 4 juillet 1898, il observa bien que, chez les moustiques, des sporozoïtes infectaient « une grosse glande du cou ». Mais, à cette époque, il ignorait la nature et la fonction de cette glande. Lorsqu'il s'aperçut qu'il s'agissait des glandes salivaires, Ross comprit immédiatement le mécanisme de la transmission. Encore fallait-il réussir à infecter des oiseaux sains. Ce qu'il réalisa en juin-juillet 1898; d'abord sur 4 moineaux et 1 tisserand; ses 5 oiseaux furent bien infectés et moururent peu après, leur foie et leur rate bourrés du pigment noir caractéristique. Ce fut la première démonstration du cycle d'un *Plasmodium* non humain. De nombreuses autres expériences vinrent ensuite confirmer et préciser ces observations.

Restait à statuer sur la nature des « black spores ». Ross estimait qu'il s'agissait de formes de résistance, qui, peut-être, une fois dans l'eau, pouvaient s'avérer infectantes pour les larves de moustiques, ce qui aurait permis la persistance du parasite, génération après génération, dans les populations culicidiennes. Manson et Laveran étaient également de cet avis. Il ne s'agissait en fait que de formes de dégénérescence.

Une fois l'ensemble de ses résultats présentés à une réunion de la British Medical Association le 24 juillet à Edinburgh, Ross souhaita démontrer la même chose pour le paludisme humain car, pour lui, « il n'y a pas de raison pour que l'hématozoaire humain diffère matériellement, dans son cycle évolutif, de celui des oiseaux ». Mais, recevant l'ordre de travailler sur le kala-azar, il dut abandonner une nouvelle fois ses travaux. Finalement, en mai 1899, Ross, quelque peu désabusé, démissionna de l'Indian Medical Service et quitta l'Inde.

Ronald Ross était certainement d'un caractère difficile; constamment irrité à tout propos, il se répandait en perpétuelles récriminations, maugréant contre les uns et les autres. Ses rapports avec ses collègues étrangers n’étaient donc pas toujours des plus cordiaux, mais, comme l’écrit Bynum (1999), « un siècle plus tard, il est toujours difficile d'aimer Ross, mais facile de l'admirer ».

Il faut également rappeler qu'en 1897, W. G. McCallum avait, lui aussi, observé le mode de fertilisation des *Plasmodium* des oiseaux puis de ceux de l'homme; il avait vu que les corps en croissant décrits par Laveran (les gamétocytes) s'arrondissent et commencent rapidement à exflageller, que les flagelles sont mobiles et s'approchent d'autres corpuscules ronds (les gamètes femelles) et les pénètrent.

## Liens D'intérêts

L'auteur ne déclare aucun lien d'intérêt.
